# Quantum Oscillations of Interacting Nanoscale Structural Inhomogeneities in a Domain Wall of Magnetic Stripe Domain

**DOI:** 10.1186/s11671-016-1680-6

**Published:** 2016-10-25

**Authors:** Andriy Shevchenko, Maksym Barabash

**Affiliations:** 1 G.V. Kurdymov Institute of Metal Physics, National Academy of Science of Ukraine, 36 Vernadskogo pr, 03680 Kyiv-142, Ukraine; 2 Technical Centre, National Academy of Science of Ukraine, 13 Pokrovskya str, 04070 Kyiv, Ukraine

**Keywords:** Uniaxial ferromagnetic film, Stripe domain, Domain wall, Vertical Bloch line, Effective mass, Gyrotropic bending, Quantum oscillations, Hybrid storage device

## Abstract

It was established that at low temperatures, quantum oscillations of a pair of interacting nanoscale structural inhomogeneities (vertical Bloch lines) occur in a domain wall of stripe domain in uniaxial ferromagnetic film. The effective mass of vertical Bloch line and conditions for this effect were determined. The effect can be used in the hybrid storage devices bit + q-bit.

## Background

The investigation of structural inhomogeneities in domain walls (DWs) in ferromagnetic materials is an important issue of the physics of nanoscale ferromagnetic systems. In uniaxial films, the vertical Bloch lines (VBLs) [1], or local transition zones between subdomains of DW, are often considered. These nanoscale objects with characteristic size ≤10^2^ nm are the topological elements of DW internal structure, which impact on the behavior of DW in external magnetic fields and give rise to various dynamic effects (see [1, 2]). Moreover, VBLs appear not only in DWs in ferromagnetic films but also in nanosized ferromagnetic stripes [3–5] and wires [6]. Similar topological structures were recently found out in the ferroelectric materials [7]. These examples show that the VBL can be regarded as an integral part of the process of self-organization of the order parameter in nanoscale magnetic and electrical structures.

It is worthwhile to note that a pair of VBLs with a negative topological charge in the domain wall of magnetic stripe domain (SD) was proposed to be used as a bit of information in the solid-state data storage devices [8, 9]. In this system, there are exchange and magnetostatic interactions between the vertical Bloch lines; as shown below, the resultant force causes minor oscillations of VBL. At the same time, the VBL and Bloch points (intersections of two VBLs) exhibit macroscopic quantum properties at low temperatures (T < 1 K) [10–15]. It is natural to assume that this feature of structural inhomogeneities in DW will be reflected both in the quantum dynamics of two interacting VBLs and in the properties of new data storage devices on their basis. Indeed, by superimposing magnetic field that provides quantum oscillation mode of VBL pair in the domain wall of SD, we can form a q-bit with the ground state and another level of VBL oscillation spectrum, excited by magnetic field. Therefore, there are prerequisites for creating a hybrid data storage device bit + q-bit. It should be emphasized that this situation is possible only due to the interaction between the nanoscale inhomogeneities in DW (VBLs in our case).

Thus, the study of quantum oscillations of interacting VBLs in a domain wall of SD is of great importance. A solution for this problem allows to develop ultra-dense data storage devices with high functionality, which would combine “classical” and quantum modes of data recording.

This work deals with the quantum oscillations of a pair of interacting VBLs in a domain wall of SD in uniaxial ferromagnetic film whose quality factor *Q* (the ratio of magnetic anisotropy energy to magnetostatic energy) is significantly higher than 1. In the first section, an expression for the VBL effective mass in a domain wall of SD is derived. This expression will be used in the following sections for the study of VBL quantum oscillations and conditions of their excitation.

## Methods

### The Effective Mass of VBL

Let us consider a VBL in a domain wall of SD. We will determine the effective mass of the vertical Bloch line *m*_*L*_, using the general formalism proposed in [16]. To do this, we need to find the gyrotropic bending of DW due to the motion of VBL at velocity *v*_*L*_. The problem will be solved in a Cartesian coordinate system with origin in the center of the domain, *Z* axis oriented along the axis of anisotropy, and *X* axis along the vertical Bloch line. Then, the Lagrangian L of the system can be written as

1 $$ L=\frac{2{M}_S h}{\gamma}{\displaystyle \sum_{i=1,2}{\displaystyle \int d x}{\overset{.}{q}}_i{\psi}_i}-\frac{h{\sigma}_0}{2}{\displaystyle \sum_{i=1,2}{\displaystyle \int d x}\left[{\left(\frac{\partial {q}_i}{\partial x}\right)}^2+{\varDelta}^2{\left(\frac{\partial {\psi}_i}{\partial x}\right)}^2+{Q}^{-1}{ \sin}^2{\psi}_i\right]}-{W}_m, $$

where *q*_*i*_ is the coordinate of normal displacement of DW center, *ψ*_*i*_ is the angle between the magnetization vector ${\overrightarrow{M}}_{S_i}$ in the DW center and *X* axis, *γ* is the gyromagnetic ratio, *h* is film thickness, *σ*_0_ is the surface energy of DW, *Δ* is the width of DW, and *W*_*m*_ is the magnetostatic energy of SD due to the presence of magnetic “charges” on the film surface.

To simplify the task, the domain wall of SD without VBL will be considered to be pinned by defects and fixed (the evaluation will be provided below). In this case, according to the results of [17], the energy, *W*_*m*_ can be written as

2 $$ {W}_m=16\pi {M}_S^2{\displaystyle \underset{-\infty }{\overset{+\infty }{\int }} dk{q}_k^2\left(\frac{1}{2} \ln \left(1+{\kappa}^{-2}\right)\kern0.5em - C- \ln hk/2-{K}_0(hk)\right)}, $$

where *q*_*k*_ is the Fourier transform of *q*, *κ* = *a*/*h*, *a* is the SD width, *C* = 0.5772 is Euler’s constant, *K*_0_(*hk*) is the Macdonald function, and $\varLambda =\varDelta \sqrt{Q}$ is the characteristic size of VBL.

The magnetization pattern in DW with VBL can be written as *ψ* = 2*arctg* exp(*ξ*/*Λ*), where *ξ* = *x* − *x*_*L*_ and *x*_*L*_ is the coordinate of VBL center. In this case, calculating variations, taking into account (2), Lagrangian (1) and solving the corresponding variational problem for *v*_*L*_ < *ω*_*M*_ *Λ*, we find for the DW gyrotropic bending:

3 $$ q\left(\xi \right)=\frac{v}{\omega_M\varLambda}\varDelta {\displaystyle \underset{0}{\overset{+\infty }{\int }} dk\frac{ \cos \left( k\xi /\varLambda \right) c{h}^{-1} k\pi /2}{f_k}}, $$

where

$$ {f}_k={k}^2+\frac{2\varDelta}{\pi h}\left(\frac{1}{2} \ln \left(1+{\kappa}^{-2}\right)\kern0.5em - C- \ln \frac{hk}{2\varLambda}-{K}_0\left(\frac{hk}{\varLambda}\right)\right),\kern0.5em {\omega}_M=4\pi \gamma {M}_S. $$

It is also follows from the formulas (1) and (2) that the frequency of SD free oscillations is determined by *f*_*k*_ *ω*_*M*_.

The effective mass of VBL can be found from the following equation (see [16]):

4 $$ \frac{1}{2}{F}_g{q}_{\xi \to 0}=\frac{1}{2}{m}_L{v}_L^2, $$

where *F*_*g*_ = 2*πM*_*S*_ *v*_*L*_/*γ* is the gyrotropic force acting on the DW from the moving VBL.

According to the above and using (3) and (4), one can easily find

5 $$ {m}_L=\frac{Q^{-1/2}}{2{\gamma}^2}{\displaystyle \underset{0}{\overset{+\infty }{\int }} dk\frac{c{ h}^{-1} k\pi /2}{f_k}}. $$

An analysis of the integral in (5) for typical parameters of ferromagnetic films and SD (*γ* ~ 10^7^ Oe^−1^s^−1^, *Q* ~ 10 − 16, *Δ* ~ 10^− 6^ cm, *h* ~ 10^− 4^ cm, 4*πM*_*S*_ ~ (10^2^ − 10^3^) G, and *κ* ~ 1) shows that its value is determined by the behavior of function *f*_*k*_ which has a minimum at *k*_*c*_, and ${k}_c\approx {\left({f}_0-{f}_{k_c}\right)}^{1/2}\sim \varLambda / h$, where ${f}_0={f}_{k\to 0}=\frac{\varDelta}{\pi h} \ln \left(1+{\kappa}^{-2}\right)\sim \frac{1}{\pi}{10}^{-2} \ln 2$.

Therefore, the function *f*_*k*_ can be approximated as

6 $$ {g}_k={f}_{k_c}+\left({f}_0-{f}_{k_c}\right){\left(1- k/{k}_c\right)}^2. $$

**Fig. 1:**
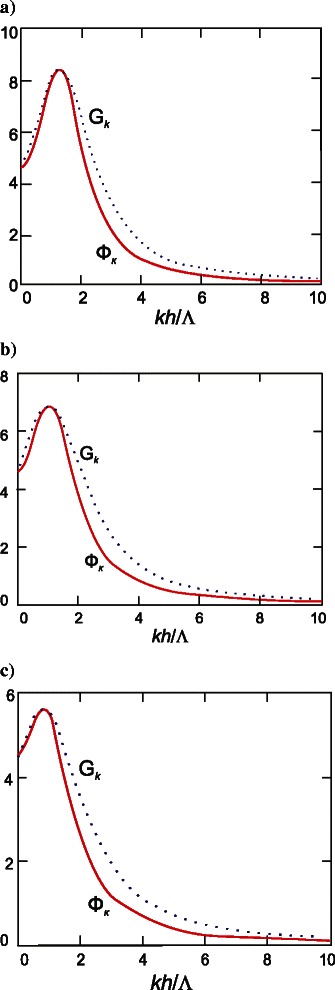
Functions *Φ*_*k*_ and *G*_*k*_ at different values of film quality factor *Q*: **a** *Q* = 10; **b** *Q* = 12; **c** *Q* = 16

The functions ${\varPhi}_k={f}_k^{-1}\varDelta / h$ and ${G}_k={g}_k^{-1}\varDelta / h$ for various values of film quality factor are plotted in Fig. 1; a good correspondence between the integrands is observed. Then, using (6) and the properties of function *f*_*k*_, we eventually find from (5):

7 $$ {m}_L=\frac{Q^{-1/2}}{2{\gamma}^2\sqrt{f_{k_c}}} arctg\frac{\sqrt{f_{k_c}}}{\sqrt{f_0-{f}_{k_c}}}. $$

It should be noted that the obtained expression also describes the effective mass of VBL in an isolated DW. In this case *κ* → ∞, and instead of the term *f*_0_ = *Δ* ln(1 + *κ* ^− 2^)/*πh* → 0 in the expression for ${f}_{k_c}$, the term *f* = *H*′*Δ*/4*πM*_*S*_ appears, where *H*′ is a gradient magnetic field which stabilizes DW. Then, ${f}_{k_c}$ is expressed through the critical field of DW bending instability *f*_*c*_: ${f}_{k_c}= f-{f}_c$ [18, 19]. It is easy to see that in the case of intense stabilizing fields with (*f* − *f*_*c*_)/*f*_*c*_ > > 1, (7) transfers into the expression for the effective mass of VBL in DW of a solid ferromagnetic material [20]:

8 $$ m{}_L=\frac{\pi}{4}{\gamma}^{-2}{Q}^{-1/2}{f}^{-1/2}. $$

In turn, it is easy to see that if *f* is close to *f*_*c*_, i.e., (*f* − *f*_*c*_)/*f*_*c*_ < < 1, then the function ${f}_k^{-1}$ has a sharp peak at *k* = *k*_*c*_. Therefore, integrating (5) with the integrand ${g}_k^{-1}$ near this point, we obtain an expression that matches well with the formula for the effective mass of VBL in [16, 19]:

9 $$ {m}_L=\frac{\pi}{2}{\gamma}^{-2}{Q}^{-1/2}{\left( f-{f}_c\right)}^{-1/2}. $$

Note that the agreement of (8) and (9) with the known expressions for *m*_*L*_ evidences the correction of proposed approach to the determination of VBL effective mass. Besides, expression (7) shows that the VBL effective mass is determined by the structure of spectrum of domain oscillations.

Let us now consider the pinning of domain wall in SD without VBL by defects. The opposing DW acts on the unit area of DW with magnetostatic attractive force ${F}_{m_{1,2}}$, causing its displacement *q*_2_. In turn, given that the surface density of magnetic charge equals *M*_*S*_, we can estimate magnetostatic energy of interaction between two DWs ${W}_{m_{1,2}}$ as ${W}_{m_{1,2}}\sim {\varLambda}^2{q}_1{q}_2{M}_S^2/ a$, where *q*_1_ is determined by (3). Then, we find

$$ {F}_{m_{1,2}}=\frac{1}{h\varLambda}\frac{\delta {W}_{m_{1,2}}}{\delta {q}_2}\sim \frac{\varDelta}{h}\left(\frac{v_L}{\omega_M\varLambda}\right){M}_S^2 $$

Comparing this expression with the force *F*_*d*_ ~ 2*M*_*S*_ *H*_*c*_ (*H*_*c*_ ~ 0.1 Oe is the coercitivity) that acts on the DW from defects, we obtain the condition under which the DW movement can be neglected:

$$ {v}_L/{\omega}_M\varLambda <<2{M}_S^{-1}{H}_c h/\varDelta \sim {10}^{-1}-1. $$

Obviously, this relation is consistent with the previously mentioned requirement for the velocity of vertical Bloch line *v*_*L*_ (see the derivation of (3)).

### Problem Solving and Discussion

Consider a pair of VBLs with the same topological charge in a domain wall of SD. There is an exchange interaction between spins of each VBL, and the energy of this interaction *W*_*e*_ is determined as [1].

$$ {W}_e= A{\displaystyle \int dxdy{ \sin}^2\theta {\left(\partial \psi /\partial x\right)}^2=4 A\varDelta /\varLambda}, $$

where *A* ~ 10^− 7^ erg/cm is the exchange constant and *θ* and *ψ* are the polar and azimuth distributions of magnetization vector, respectively, which determine the internal structure of DW and VBL.

In turn, *W*_*e*_ can be written as

$$ {W}_e\sim {e^{\ast}}^2/\varLambda, $$

where *e* ^∗2^ is the effective electron density of interacting VBL spins per unit length.

Equating the above expressions, we obtain *e* ^∗2^ = 4*AΔ*. Considering that the effective electron density of the whole VBL equals 2*e* ^∗2^, we find the exchange energy of two VBLs separated by distance *s*:

$$ {W}_e=16\frac{A\varDelta}{\varLambda} \exp \left(- s/\varLambda \right). $$

Differentiating *W*_*e*_ by *s*, we find the force of exchange interaction of VBL per unit length:

10 $$ {F}_e=\frac{16 A}{\varLambda \sqrt{Q}} \exp \left(- s/\varLambda \right). $$

In addition to the repulsive force *F*_*e*_, there is an attractive magnetostatic force *F*_*m*_ between two VBLs, that can be expressed as [1].

11 $$ {F}_m=8\left(\pi \varDelta {M}_S^2\right)/ s. $$

Comparing these two forces, we get the well-known equation for the equilibrium distance *s*_0_ between vertical Bloch lines:

12 $$ s{}_0/\varLambda = \ln \left(4{s}_0\sqrt{Q}/\pi \varLambda \right), $$

This equation has the following solution: ${s}_0=\sqrt{2}\pi \varLambda$ [21].

Considering the VBLs as quasiparticles, examine now the small fluctuation displacements of VBL from the equilibrium position. Obviously, these displacements are equal in magnitude and opposite in phase. During movement along *X* axis, a VBL experience force *F* = *F*_*e*_ − *F*_*m*_. According to (10)–(12), this force can be written as

13 $$ F=-\frac{\pi}{2\varLambda}{E}_L{Q}^{-1/2}\left(1-\varLambda /{s}_0\right)\frac{2\delta s}{s_0}, $$

where *E*_*L*_ = 8*AQ* ^− 1/2^ is the energy of VBL, normalized by length, and *δs*/*s*_0_ < < 1 is the displacement of VBL. Obviously, for the VBL moving in opposite direction *F* > 0. Approaching each other, VBLs can be considered in the same manner.

Thus, small displacements of interacting VBLs from the equilibrium position cause the force *F* = *F*_*e*_ − *F*_*m*_ that is directed opposite to their displacements. This force has stiffness factor *k*_*L*_ = − ∂*F*/∂*δs* (per unit length); therefore, using Eqs. (7), (12), and (13) and formula *ω*_*L*_ = (*k*_*L*_/*m*_*L*_)^1/2^, we can find the frequency of VBL small oscillations:

14 $$ {\omega}_{BL}=\frac{\omega_M{Q}^{-1/4}}{\pi}{\left[\frac{\left(\sqrt{2}\pi -1\right)\sqrt{f_{k_c}}}{arctg\left(\sqrt{f_{k_c}}/\sqrt{f_0-{f}_{k_c}}\right)}\right]}^{1/2}. $$

Estimation of the VBL effective mass and the oscillation frequency for the films with a quality factor *Q* = 10 − 16 gives values of *m*_*L*_ ≈ (3.8 − 3.3) ⋅ 10^− 15^ g/cm and *ω*_*L*_ ~ (2.1 − 1.8) ⋅ 10^− 1^ *ω*_*M*_, respectively. As expected, these values reduce with increasing film quality factor. Indeed, according to (12), higher *Q* leads to a longer equilibrium distance *s*_0_. As a result, the values of VBL interaction energy and DW gyrotropic bending decrease, which ultimately leads to reduction of *ω*_*L*_ and *m*_*L*_.

Consider now the possibility of quantum oscillations of a pair of VBLs. Let us compare the average energy of vertical Bloch line ${\overline{W}}_{H, L}=2{\pi}^2{\varDelta}^2{M}_S^2{\left({H}_x\right)}^2 h/{m}_L{\omega}_L^2$ in uniform magnetic field *H*_*x*_ directed along the axis of its oscillations with the “interstate” energy gap *ΔW*_*L*_ = *ℏω*_*L*_ (where *ℏ* is the Planck constant) (see [15, 22]). Using (7) and (14), after some transformations, we find that *n* > > 1 (i.e., quantum transitions from the ground level to quasiclassical zone are considered), and so ${\overline{W}}_{H, L}>>\varDelta {W}_L$ if the external fields satisfy inequation

15 $$ {h}_x>>{\left[\left(\frac{\sqrt{2}\pi -1}{2\pi}\right)\left(\frac{\varDelta}{h}\right)\varDelta {W}_L/{\sigma}_0{\varDelta}^2\right]}^{1/2}, $$

where *h*_*x*_ = *H*_*x*_/8*M*_*S*_.

It should be noted that small oscillations of vertical Bloch line in a domain wall of magnetic bubble were studied in [15]. However, the stiffness factor of these oscillations was not associated with the interaction between vertical Bloch lines and was provided by magnetic field applied normally to VBL.

Estimation of (15) at *σ*_0_ ~ 1 erg/cm^2^ gives *h*_*x*_ > > 10^− 4^ that is consistent with the requirement for planar magnetic fields applied to DW.

It is worthwhile to note that *w*_*n*_ is the probability distribution *ñ* = *W*_*H*,*L*_/2*ℏω*_*L*_ of quanta on *n* discrete levels of VBL spectrum, which is determined by Poisson distribution [22]: ${w}_n=\frac{1}{n!}{e}^{-\tilde{n}}{\tilde{n}}^n$. As follows from the calculations, *n* ~ 10 is a typical quantum level excited by magnetic field *H*_*x*_.

## Results and Discussion

Let us estimate the influence of dissipative force *F*_*r*_ on the process of VBL quantum oscillations in a stripe domain. Since the problem is considered in quasiclassical approximation, we can use for *F*_*r*_ the expression obtained by integrating the density of Thiele’s dissipative force [23]. In this case${F}_r=\frac{4{M}_S\alpha hv}{\gamma \sqrt{Q}}$,where *α* ~ 10^− 3^ − 10^− 1^ is the magnetization vector decay parameter.

Taking into account that the VBL velocity can be represented as *v*_*L*_ = *ω*_*L*_ *A*_*n*_, where ${A}_n=\sqrt{\left(2 n+1\right)\hslash /{m}_L{\omega}_L h}$ is a “quasiclassical” amplitude of VBL oscillations [15], the expression for *F*_*r*_ can be rewritten as

16 $$ {F}_r=\frac{4\alpha {M}_S{\omega}_L}{\gamma {Q}^{1/2}}\sqrt{\left(2 n+1\right)\hslash /{m}_L{\omega}_L h}. $$

In turn, the force *F*_*H*_ acting on VBL from magnetic field can be written as [1].

17 $$ {F}_H=2\pi \varDelta {M}_S{H}_x. $$

Using expressions (16) and (17), as well as the numerical parameters of the system (see above), one can easily find that *F*_*r*_/*F*_*H*_ < < 1 in the following fields

$$ {h}_x>>\alpha {\left(2/\pi \right)}^{1/2}{Q}^{-1/4}{\left({m}_L{\gamma}^2\right)}^{-1/2}\sqrt{E_n/{E}_L h}\sim \left({10}^{-4}-{10}^{-3}\right)\alpha, $$

where *E*_*n*_ = *ℏω*_*L*_(*n* + 1/2) is the energy of VBL quantum oscillations.

It is obvious that the obtained relation is in line with the estimation for the field *h*_*x*_ (see expression (15)) which provides quantum transitions into the quasiclassical zone of VBL spectrum. Due to various ranges of fields *h*_*x*_ required for the activation of VBL energy levels and its displacement along the DW of stripe domain (this occurs at *h*_*x*_ < 1), it is feasible to control both quantum and “classical” process of data recording in the data storage devices based on VBL.

It is natural to expect that the quantum-mechanical behavior of vertical Bloch lines will be reflected in the dynamics of DW gyrotropic bending. This problem was studied by us in [15, 24] for small oscillations of Bloch point and vertical Bloch line stabilized by magnetic field in a domain wall of magnetic bubble, where the quantum nature of the change of DW gyrotropic bending was established. Obviously, this effect should also appear in the oscillations of interacting vertical Bloch lines in a DW of stripe domain. Therefore, basing on the results of the abovementioned works, we can write for the DW gyrotropic bending the following expression

18 $$ {q}_n\sim {\left({m}_L{\gamma}^2\right)}^{1/2}\sqrt{E_n/{E}_L h}{Q}^{-1/4}\varLambda, $$

An estimation gives *q*_*n*_ ~ 10^− 3^ *Δ*. Besides, an analysis of function *f*_*k*_ and expressions (7), (14), and (18) show that at *h*/*Δ* → ∞, *k*_*c*_ ~ (*Δ*/*h*)^1/2^, *m*_*L*_ ~ (*h*/*Δ*)^1/2^, *ω*_*L*_ ~ (*Δ*/*h*)^1/4^, and *q*_*n*_ ~ (*Δ*/*h*)^5/8^, i.e., the considered effect takes place only in magnetic films and is absent in solid ferromagnetic materials.

It is easy to see that the quantization of DW gyrotropic bending is most pronounced in the ferromagnets which have DWs with high *Δ* values, such as yttrium-iron garnet (YIG) films, where *Δ* can reach up to 10^− 4^ cm [25].

It should be noted that the above values, *q*_*n*_ < < *Λ* indicate a negligible contribution of transverse component into the VBL effective mass [26]. This component *m*_*L*_ of gyrotropic origin is significant for DWs with bending comparable to the length of vertical Bloch line.

Using relation *ℏω*_*L*_ ~ *nk*_*B*_ *T* (where *k*_*B*_ is Boltzmann constant), (14), and the abovementioned numerical parameters for film and domain, we can find the temperature *T* of the process:

$$ T\sim \left({10}^{-3}-1\right) K. $$

The *T* values are in the same range with the temperatures of other quantum phenomena that occur for the vertical Bloch lines and Bloch points (see [11–15, 24]). Therefore, one can conclude that the macroscopic quantum effects in the domain systems with complex internal structure become apparent at subhelium temperatures. This allows neglecting the contribution of exchange relaxation [27] into the processes of magnetization dissipation, which accompanies these phenomena (see [15]).

## Conclusions

The effective mass of vertical Bloch line in a domain wall of a stripe domain in magnetic film with a strong uniaxial magnetic anisotropy was determined. It was found that the effective mass of vertical Bloch line is determined by the characteristics of the spectrum of domain oscillations.

The energy spectrum of low-temperature quantum oscillations of two interacting vertical Bloch lines in a domain wall of magnetic stripe domain was determined. This result can stimulate the development of a new type of hybrid memory devices which combine two recording media: specified stable states of physical memory element and quantum levels of energy spectrum of this element, activated by external field.

## Abbreviations

DWs: Domain walls
SD: Stripe domain
VBLs: Vertical Bloch lines
YIG: Yttrium-iron garnet
