# Erratum to: Quantum Oscillations of Interacting Nanoscale Structural Inhomogeneities in a Domain Wall of Magnetic Stripe Domain

**DOI:** 10.1186/s11671-017-1928-9

**Published:** 2017-03-07

**Authors:** Andriy Shevchenko, Maksym Barabash

**Affiliations:** 1 G.V. Kurdymov Institute of Metal Physics, National Academy of Science of Ukraine, 36 Vernadskogo pr, 03680 Kyiv-142, Ukraine; 2 Technical Centre, National Academy of Science of Ukraine, 13 Pokrovskya str, 04070 Kyiv, Ukraine

## Erratum

The original publication [1] contains an incorrect reference and an error in a calculation.

Reference 17 should contain the following information: “V.L.Dorman, V.L.Sobolev, A.B.Shevchenko Izgibnie kolebaniya izolirivannogo polosovogo domena v peremennih vneshnih magnitnyh polyah, Fizika metallov i metalovedenie, 67, № 4, s. 669–675. (1989).”

The calculation should be: “In this case, calculating variations, taking into account (2), Lagrangian (1) and solving the corresponding variational problem for velocity ${\overset{.}{x}}_L={v}_L<{\omega}_M\Lambda$, we find for the DW gyrotropic bending:

3 $$ q\left(\xi \right)=\frac{v_L}{\omega_M\Lambda}\Delta {\displaystyle \underset{0}{\overset{+\infty }{\int }} dk\frac{ \cos \left( k\xi /\Lambda \right) c{h}^{-1} k\pi /2}{f_k}}, $$”
